# Development of the Intrinsic Language Network in Preschool Children from Ages 3 to 5 Years

**DOI:** 10.1371/journal.pone.0165802

**Published:** 2016-11-03

**Authors:** Yaqiong Xiao, Jens Brauer, Mark Lauckner, Hongchang Zhai, Fucang Jia, Daniel S. Margulies, Angela D. Friederici

**Affiliations:** 1 Max Planck Institute for Human Cognitive and Brain Sciences, Department of Neuropsychology, Leipzig, Germany; 2 Max Planck Research Group for Neuroanatomy & Connectivity, Max Planck Institute for Human Cognitive and Brain Sciences, Leipzig, Germany; 3 College of Education, Guangzhou University, Guangzhou, R.P. China; 4 Shenzhen Institutes of Advanced Technology, Chinese Academy of Sciences, Shenzhen, R.P. China; Institute of Psychology, Chinese Academy of Sciences, CHINA

## Abstract

Resting state studies of spontaneous fluctuations in the functional magnetic resonance imaging (fMRI) blood oxygen level dependent signal have shown great potential in mapping the intrinsic functional connectivity of the human brain underlying cognitive functions. The aim of the present study was to explore the developmental changes in functional networks of the developing human brain exemplified with the language network in typically developing preschool children. To this end, resting-sate fMRI data were obtained from native Chinese children at ages of 3 and 5 years, 15 in each age group. Resting-state functional connectivity (RSFC) was analyzed for four regions of interest; these are the left and right anterior superior temporal gyrus (aSTG), left posterior superior temporal gyrus (pSTG), and left inferior frontal gyrus (IFG). The comparison of these RSFC maps between 3- and 5-year-olds revealed that RSFC decreases in the right aSTG and increases in the left hemisphere between aSTG seed and IFG, between pSTG seed and IFG, as well as between IFG seed and posterior superior temporal sulcus. In a subsequent analysis, functional asymmetry of the language network seeding in aSTG, pSTG and IFG was further investigated. The results showed an increase of left lateralization in both RSFC of pSTG and of IFG from ages 3 to 5 years. The IFG showed a leftward lateralized trend in 3-year-olds, while pSTG demonstrated rightward asymmetry in 5-year-olds. These findings suggest clear developmental trajectories of the language network between 3- and 5-year-olds revealed as a function of age, characterized by increasing long-range connections and dynamic hemispheric lateralization with age. Our study provides new insights into the developmental changes of a well-established functional network in young children and also offers a basis for future cross-culture and cross-age studies of the resting-state language network.

## Introduction

Since the seminal study of Biswal et al. [1] resting-state functional magnetic resonance imaging (rs-fMRI) has proven its great potential in the study of the intrinsic neural basis that underlies human cognitive systems. Spontaneous low-frequency (< 0.1 Hz) fluctuations (LFFs) in the human brain during resting state have shown ample evidence for intrinsic brain connectivity and functional networks in adults (e.g.,[2–5]). In recent years, the development of resting-state functional network has gathered attention and several studies have contributed findings in children (e.g., [6–8])) and even in infants [9–13].

To date, LFFs analysis has featured several functional networks which are associated with corresponding cognitive functions. One LFFs network that has been identified in rs-fMRI data (e.g., [14]) and in task fMRI data (e.g., [15]) and which is also well described in its structural neuroanatomy is the language network (for a review, see [16]). The functional organization of the language network has been described in resting-state functional connectivity (RSFC) [14, 17–21]. Tomasi and Volkow [14] showed a reproducible language network using a large rs-fMRI data set from 970 healthy subjects and seeding in Broca’s and Wernicke’s area, which is characterized by predominant short-range functional connectivity in the whole brain except for predominance of long-range connectivity in the posterior Wernicke’s area. Zhu et al. [21] further demonstrated the temporal reliability of the language network, and observed that Broca’s area was leftward lateralized while the Wernicke’s area was mainly rightward lateralized. In addition, a study analyzed LFFs from language-related fMRI experiments and showed a general framework for language processing in adults [15]. Furthermore, the development of the language network has been investigated in both task-based fMRI data (e.g., [22, 23]) and LFFs analysis of task-based fMRI data that suggests a dominance of interhemispheric connectivity at birth and childhood in contrast to a clear intrahemispheric fronto-temporal connectivity in adults [24, 25]. These findings indicate changes of the language network as a function of age, however, still little known about the developmental trajectory of the intrinsic language network in children, in general and in non-Indoeuropean languages in particular.

With the application of the fMRI technique, it has become feasible to investigate the neural basis of language acquisition and processing, even in young infants (for a review, see [26]). For example, Dehaene-Lambertz et al. [27] observed brain activations in posterior superior temporal and inferior frontal cortices for speech perception in 3-month-old infants. In adults, several regions mainly in left frontal and temporal cortices have been shown to be related to language processing; specifically, the superior temporal cortex is associated with speech perception [28, 29] and superior temporal and inferior frontal cortices engage in language comprehension (for reviews, see [16, 30]).

It has been widely acknowledged that language processing is based on both ventral and dorsal processing streams [30–34] and their underlying white matter pathways [23, 35, 36]. Within the left hemisphere the ventral pathway connecting anterior superior temporal gyrus (aSTG) and inferior frontal gyrus (IFG, BA 44/45) is assumed to support sound-to-meaning mapping and local syntactic structure building, whereas dorsal pathways connecting posterior superior temporal gyrus and sulcus (pSTG/STS) and IFG support auditory-to-motor mapping and the processing of sentential syntax (for a review, see [30]).

As a powerful tool to investigate the spontaneous neuronal activity, rs-fMRI has great advantages in terms of acquiring data from young children who are not able to perform complicated tasks in the scanner, and characterizing functional brain networks independent of specific tasks. Therefore, the rs-fMRI technique is a sound solution to sketch the picture of the development of functional networks of the human brain. It avoids the bias in specific task selection and experimental design on the one hand, and allows exploring the general development at a network level on the other hand. Here, we selected the functional network of language-relevant brain regions because this network is well established and comprises of brain areas across lobes and hemispheres. It has been well-known that language ability develops very fast during the first years of life, and the underlying neural basis might be somewhat unveiled in respect of the language network development with age in early life.

Considerable amount of evidence from language fMRI studies has shown the presence of language lateralization in children and its dynamic shift with age associated with developing language skills in children and adolescents [37]. Szaflarski et al. [38] examined the effect of age on language lateralization using a verb generation task, and observed increasing left lateralization between the ages 5 and 20 years, plateauing between 20 and 25 years, and decreasing between 25 and 70 years. With regard to the left language lateralization in adults, coincidently, it was also observed in the resting-state language network as reported in recent rs-fMRI studies across different languages such as English, German, Dutch and Chinese [14, 21]. The process of language lateralization with language development in children, however, triggers a still uncovered question—how does the lateralization of the resting-state language network develop with age?

In the present study, RSFC of the language network was investigated in typically developing Chinese preschool children at ages 3 and 5 years given the rapid development during this time. Fifteen rs-fMRI data sets in each age group were acquired while children were in a natural sleep state. Intrinsic connectivity analysis was conducted with four language-related regions of interest (ROIs), including the left aSTG, left pSTG, right aSTG and left IFG, which had been shown to be activated in adult Chinese native speakers and chosen as ROIs in dynamic causal modeling [39]. Expecting to detect age-related changes at the network level, here, we firstly explored the development of intrinsic connectivity in the language network based on these selected ROIs. Furthermore, we investigated the functional asymmetry of the intrinsic language network and its development by seeding in aSTG, pSTG and IFG. Based on previous studies, we expected to observe the presence of hemispheric asymmetry in the functional connectivity of these regions and also an increase of left lateralization with age.

## Subjects and Methods

### Participants

Thirty-six typically developing preschool children aged 3 and 5 years participated in this study, including 19 3-year-olds (10 boys, 36±2 months) and 17 5-year-olds (9 boys, 60±2 months), as reported in a separate analysis [40]. All children were recruited from kindergartens in Nanshan District, Shenzhen. They were right-handed, monolingual Chinese speakers with no history of neurological, medical, or psychological disorders. The parents of these children gave written informed consent, and children gave verbal assent prior to participation. Before MRI scanning, all children were examined by China-Binet intelligence test, scoring between 90 and 110 (3-year-olds: 94.00 ± 2.62; 5-year-olds: 97.40 ± 6.48). The study was approved by the Institutional Review Board of Shenzhen Institutes of Advanced Technology, Chinese Academy of Sciences.

All children were scanned during natural sleep. In order to ensure that children could easily fall asleep, MRI scanning was performed in the afternoons or evenings, and the day before the scanning, they were asked to sleep later in the evening and to get up earlier in the morning than usual. Children were carried into the MRI scanner after falling asleep in the waiting room, and they were accompanied by their parents and one of the experimenters during the course of scanning. The process would be terminated if they woke up. Finally, MRI data were acquired from 30 children, 15 in each age group (3-year-olds: 8 boys, IQ: 94.50 ± 2.53; 5-year-olds: 7 boys, IQ: 97.2 ± 6.24).

### Data acquisition

The MRI data acquisition was performed on a 3 Tesla Siemens MRI scanner (Siemens Magnetom Tim Trio) with a 12-channel head coil in Shenzhen Institutes of Advanced Technology, Chinese Academy of Sciences. Functional images were acquired using a T2*-weighted single-shot echo planar imaging (EPI) sequence (TR/TE = 2500 ms/30 ms, flip angle = 90°, slice thickness = 2.5 mm, gap = 0.5 mm, FOV = 200 mm×200 mm, matrix = 64×64, 36 slices, 192 volumes). After the functional scanning, T1-weighted 3D structural images were obtained with magnetization prepared rapid gradient echo (MPRAGE) sequence (TR/TE = 1900 ms/2.53 ms, flip angle = 9°; slice thickness = 1.0 mm, gap = 0 mm, FOV = 250 mm×250 mm, matrix = 250×250). The whole scanning process lasted 15 minutes. For more details on the data acquisition protocol, see Xiao et al. [40].

### Image preprocessing

Before image preprocessing, the first seven EPI volumes were discarded to allow for signal equilibration, and the remaining 185 volumes were included in the analysis. We used the preprocessing procedure employed in a previous study (for details see [40]), including: *i)* slice timing; *ii)* head motion correction using a least squares approach and a 6 parameter (rigid body) spatial transformation; *iii)* producing group-averaged templates via new segment and the diffeomorphic anatomical registration through exponentiated lie algebra (DARTEL) [41]. Each group-averaged template was firstly normalized to Montreal Neurological Institute (MNI) space with 8 mm full-width at half-maximum (FWHM) isotropic Gaussian kernel, and then functional images of each group were normalized to the corresponding template and resampled to voxel size 2*2*2 mm. Subsequently, normalized functional images were smoothed with 8 mm FWHM kernel. *iv)* a component based noise reduction method (CompCor) was performed using the Functional Connectivity Toolbox (CONN) [42] (http://web.mit.edu/swg/software.html) to correct for physiological noise by regressing out principal components (PC) from noise ROIs (i.e., white matter mask and cerebrospinal fluid mask from the aforementioned new segmentation procedure) [43], including temporal despiking and nuisance regression (12 head motion parameters, whiter matter mask and cerebrospinal fluid mask; three PC parameters for each mask); *v)* band-pass filtering (0.01–0.1 Hz).

In order to further reduce the effect of head motion, framewise displacement (FD) was calculated for each participant following Jenkinson et al. [44] as suggested by Yan et al. [45]. The mean FD was controlled as a covariate of no interest in the group-level statistical analyses, although it did not show statistical difference between 3-year-olds (M±SD: 0.085±0.047 mm) and 5-year-olds (M±SD: 0.1±0.097 mm) in the final sample (*t*(28) = -0.55, *p* = 0.59).

### Resting-state functional connectivity analysis

**Fig 1:**
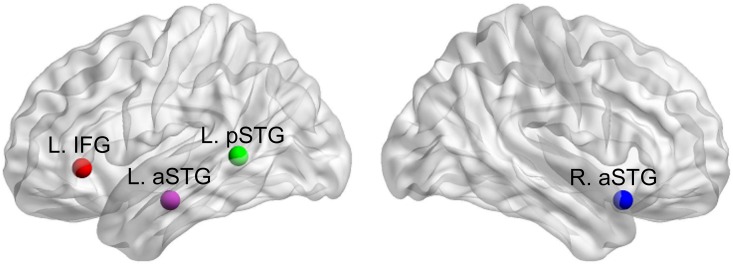
The depiction of regions of interest in Table 1. L., left hemisphere; R., right hemisphere. aSTG, anterior superior temporal gyrus; pSTG, posterior superior temporal gyrus; IFG, pars triangularis of inferior frontal gyrus.

In this analysis, four ROIs were selected based on a previous language study [39]. These regions indicated brain activations for speech intelligibility effect (intelligible > unintelligible) in the Chinese group. In this study, the intelligible stimuli were idiomatic word pairs and unintelligible counterparts were time-reversed of intelligible stimuli by removing the intelligibility of the forward speech but preserving the acoustic and voice identity information. The details of these ROIs are shown in Table 1 and Fig 1.

**Table 1:** Regions of interest for language network [39].

Region	BA	Abbreviation	MNI coordinates		
			*x*	*y*	*z*
L. anterior superior temporal gyrus	38	L. aSTG	-50	14	-18
L. posterior superior temporal gyrus	22	L. pSTG	-60	-48	4
R. anterior superior temporal gyrus	38	R. aSTG	48	16	-18
L. pars triangularis of inferior frontal gyrus	45	L. IFG	-48	30	-2

Note: L., left hemisphere; R., right hemisphere.

RSFC analysis was performed by using REST software [46], (http://www.restfmri.net), and voxel-wise functional connectivity was calculated for each ROI. Specifically, the mean time series of each seed were first computed for each participant by averaging the time series of all the voxels in the ROI (a sphere with 6 mm radius), and then the Pearson’s correlation coefficients between the time series of each seed and time series of all other regions within the whole brain were calculated. The individual level RSFC correlation map (*r*-map) was obtained for each subject, and subsequently, all *r*-maps were converted into *z*-maps with application of Fisher’s *r*-to-*z* transformation to get approximately normally distributed values for further statistical analysis.

One-sample *t*-test was carried out to obtain the group-level functional connectivity map for each ROI, controlling for age, gender as well as mean FD. Results were corrected at the cluster-level using Gaussian random field (GRF) theory (|*Z*| ≥ 3.3, voxel-wise *p* < .001, cluster-wise *p* < .05, GRF corrected). Two-sample *t*-tests were performed for the comparison between 3- and 5-year-olds, controlling for gender and mean FD. Results were corrected at the cluster-level using GRF theory (|*Z|* ≥ 2.58, voxel-wise *p* < .01, cluster-wise *p* < .05, GRF corrected). Finally, all the maps were visualized with the BrainNet Viewer [47], (http://www.nitrc.org/projects/bnv/).

### Hemispheric asymmetry analysis

In order to assess hemispheric asymmetries, preprocessed data were normalized to a symmetric template prior to functional asymmetry analysis. Firstly, a mean template was created based on the normalized T1 template for each age group. Secondly, a group-specific symmetrical template was generated by averaging the mean template with its left-right mirrored version. Thirdly, normalized T1 images were nonlinear registrated to MNI space for each subject using the group-specific symmetric template. Finally, the transformations were applied to each subject’s functional data.

The functional asymmetry of the language network was evaluated based on predefined left hemispheric ROIs. The left-right-flipped (LR-flipped) regions of these ROIs were defined as right aSTG, right pSTG, as well as right IFG, separately. RSFC maps of these regions were produced using the same procedure as aforementioned. The asymmetry of RSFC in the present study was defined in a same manner as shown in previous studies [21, 48]. Firstly, new sets of z-maps were generated by LR-flipping the individual z-maps of right aSTG, right pSTG and right IFG. Subsequently, the z-transformed RSFC maps of left ROIs deducted LR-flipped z-transformed RSFC maps of right ROIs to obtain the asymmetry index (AI), which can be shown as the equation [21]:

$$AI=zFC_{L}-\;zFC_{flipped\;R}$$

AI maps of all ROIs were calculated voxel by voxel across subjects for both 3- and 5-year-olds. One-sample *t*-tests were performed to reveal regions which showed significant hemispheric asymmetry. More specifically, the left hemisphere of the *t*-maps represents the asymmetry of RSFC of left ROIs with the ipsilateral hemisphere (e.g., differences between connectivity of left aSTG in the left hemisphere and that of right aSTG in the right hemisphere), and the right hemisphere of the *t*-maps demonstrated the asymmetry of connectivity of left ROIs with the contralateral hemisphere (e.g., differences between left aSTG seed—right hemisphere connectivity and right aSTG seed—left hemisphere connectivity).

Moreover, in order to investigate the hemispheric asymmetry changes with age, group comparison between AI maps from 3- and 5-year-olds was carried out for each seed using two-sample *t*-tests. Finally, all the resultant *t*-maps were corrected for multiple comparison using GRF theory (|*Z|* ≥ 2.58, voxel-wise *p* < .01, cluster-wise *p* < .05, GRF corrected).

## Results

### The language network in each age group

**Fig 2:**
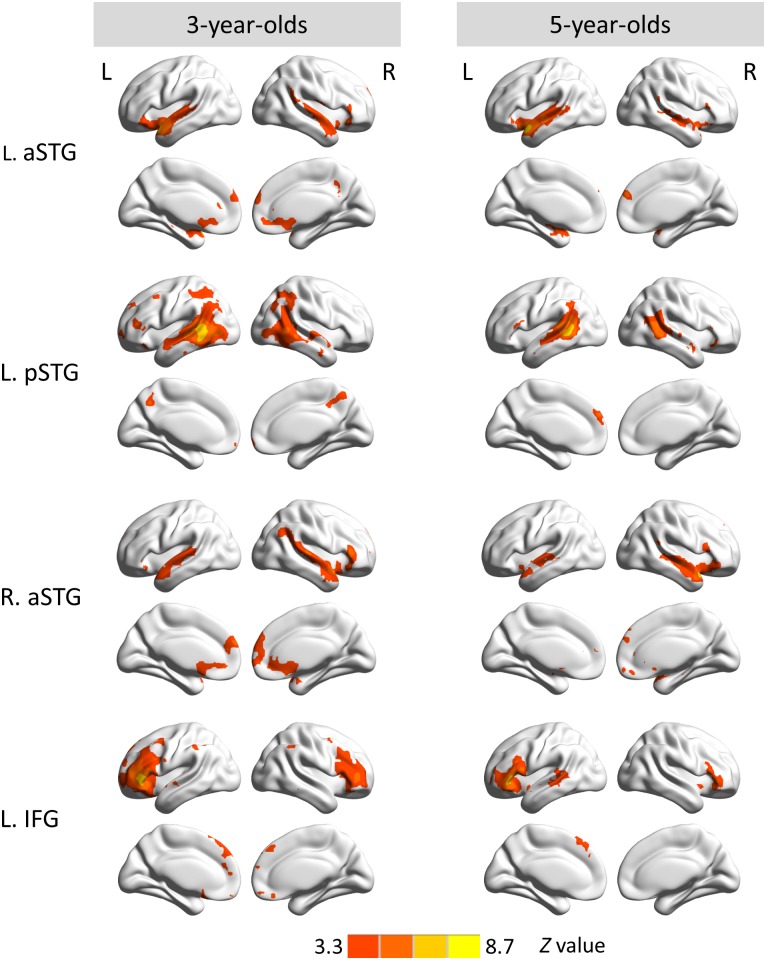
Functional connectivity maps seeding in the left aSTG (first row), left pSTG (second row), right aSTG (third row) and left IFG (last row) in 3- and 5-year-olds. The red-yellow color bar indicates significant positive connections with the seed. Maps display voxels showing significant correlations (*Z* ≥ 3.3, voxel-wise *p* < .001, cluster-wise *p* < .05, GRF corrected). L., left hemisphere; R., right hemisphere.

Functional connectivity maps seeding in the left aSTG, left pSTG, right aSTG and left IFG revealed similar distributed patterns of connections in both age groups (Fig 2). Left aSTG RSFC, in 3-year-olds, showed positive connectivity bilaterally with temporal cortices, IFG, anterior cingulate cortex (ACC), posterior cingulate cortex/precuneus (PCC), and ventral and dorsal medial prefrontal cortex (vMPFC/dMPFC); in 5-year-olds, it demonstrated positive connectivity bilaterally with temporal cortices, IFG, and dMPFC. For the left pSTG RSFC, in 3-year-olds, correlations were mainly observed in bilateral temporal cortices, inferior parietal lobe (IPL), middle frontal gyrus (MFG), IFG, vMPFC as well as PCC, while in 5-year-olds correlations with bilateral temporal cortices, dMPFC, and left IFG (BA 44) were found. For right aSTG RSFC, children at the age of 3 years showed correlations mainly in bilateral temporal cortices, ACC, dMPFC, and right IFG, whereas children at the age of 5 years showed correlations in bilateral temporal cortices, right IFG, vMPFC and dMPFC. Regarding the left IFG RSFC, 3-year-old children showed correlations to bilateral IFG, dMPFC, IPL, and left anterior temporal cortex, whereas in 5-year-old children, correlations mainly covered bilateral IFG, dMPFC and left pSTG.

### Changes in the language network from ages 3 to 5 years

**Fig 3:**
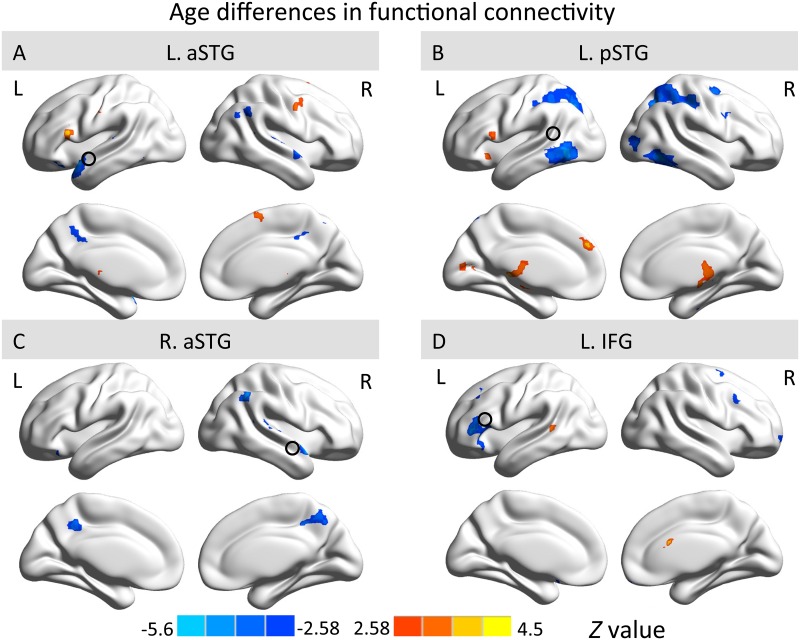
Comparison of functional connectivity maps between 3- and 5-year-olds in left aSTG (A), left pSTG (B), right aSTG (C) and left IFG (D). The red-yellow color bar indicates stronger connectivity in 5-year-olds, while the blue color bar indicates stronger connectivity in 3-year-olds. Maps display voxels showing significant differences between two age groups (|*Z*| ≥ 2.58, voxel-wise *p* < .01, cluster-wise *p* < .05, GRF corrected). L., left hemisphere; R., right hemisphere.

For the direct comparison between both age groups, two-sample *t*-tests showed developmental changes in children from ages 3 to 5 years (|*Z*| ≥ 2.58, voxel-wise *p* < .01, cluster-wise *p* < .05, GRF corrected) (Fig 3). The connected regions to each seed identified from the comparison are shown in Table 2. When seeded in the right aSTG, 3-year-olds showed greater connectivity with the left inferior orbital frontal gyrus, right superior temporal lobe, right angular gyrus and IPL, as well as bilateral PCC. Meanwhile, we consistently observed stronger connectivity between left IFG and superior temporal cortex in 5-year-olds when seeded in the left aSTG, left pSTG and left IFG. This holds for the RSFC between left aSTG seed and left IFG (BA 44), between left pSTG seed and left IFG (BA 44), and between left IFG seed and left pSTS (BA 22).

**Table 2:** Comparison of functional connectivity maps between 3- and 5-year-olds.

Seed	Region	BA	Peak MNI coordinates			Voxels	*Z* value
			*x*	*y*	*z*		
L. IFG IIFGaSTG	L. postcentral gyrus	3	-44	-18	36	150	3.7
	L. inferior frontal gyrus	44/45	-50	18	14	235	3.67
	L/R. Thalamus		12	-28	6	259	3.46
	R. precentral gyrus	4/6	54	-6	42	150	3.17
	L. superior temporal pole, inferior orbital frontal gyurs	38	-48	12	-16	1988	-5.54
	R. angular gyrus	39	58	-56	36	516	-4.52
	R. superior temporal gyrus	22	52	-4	-10	1141	-4.26
	L/R. posterior cingulate cortex/precuneus	7	-2	-48	44	1267	-3.94
	L. inferior/middle temporal gyrus	37	-44	-54	-6	199	-3.87
L. pSTG	L/R. thalamus		-8	-30	2	985	4.34
	L. dorsomedial prefrontal cortex	32	-2	48	32	397	3.86
	L. putamen, pullidum, insula, caudate		-26	16	-5	287	3.76
	R. calcarine	17	24	-54	12	130	3.68
	L. calcarine/cuneus, lingual	17	-12	-82	14	248	3.35
	L. inferior frontal gyrus	44	-50	16	12	108	3.09
	R. inferior temporal gyrus, inferior/middle occipital gyrus	37	46	-44	-10	2101	-5.27
	R. superior/inferior parietal lobe	40	24	-66	44	2664	-4.72
	L. inferior/middle temporal gyrus	37	-50	-64	-6	2059	-4.6
	L. superior/inferior parietal lobe	40	-26	-70	42	3514	-4.49
	R. superior/middle frontal gyrus	6	24	-6	54	359	-4.24
	R. inferior frontal gyrus, precentral gyrus	6	54	6	36	295	-3.77
R. aSTG	R. superior temporal pole	38	52	8	-12	1097	-4.59
	L/R. precuneus	7	-14	-42	40	1264	-4.03
	R. angular gyrus, inferior parietal lobe	40	52	-52	42	420	-3.91
	L. inferior orbital frontal gyrus	47	-36	30	-20	213	-4.11
L. IFG	R. caudate		6	16	20	134	3.89
	L. superior temporal sulcus	22	-50	-46	6	134	3.46
	L. inferior (orbital) frontal gyrus	47/45	-48	30	-4	1309	-5.03
	L/R. ventromedial prefrontal cortex		0	56	42	143	-4.32
	R. middle/superior frontal gyrus	6	24	0	72	298	-3.92
	R. middle frontal sulcus		30	38	22	143	-3.89
	L superior frontal gyrus	9	-20	30	48	758	-3.47

Note: BA, Brodmann area; L., left hemisphere; R., right hemisphere. The positive z value indicates stronger connections in 5-year-olds while the negative z value indicates stronger connections in 3-year-olds.

### Hemispheric asymmetry of the language network

**Fig 4:**
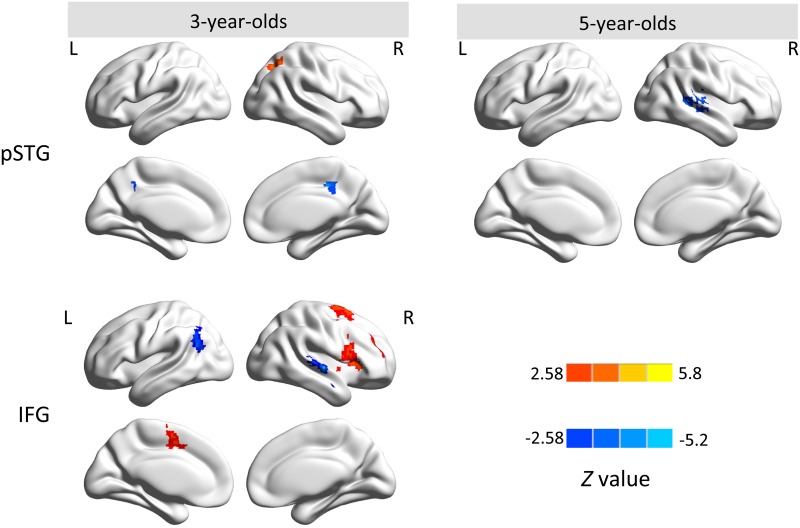
Significant hemispheric asymmetry of pSTG in both age groups (first row) and of IFG in 3-year-olds (second row). The foci of the left hemisphere show significant asymmetric functional connectivity with their ipsilateral seeds, and the foci in the right hemisphere show significant asymmetric functional connectivity with their contralateral seeds, corrected for multiple comparisons (|*Z*| ≥ 2.58, voxel-wise *p* < .01, cluster-wise *p* < .05, GRF corrected). The red-yellow color bar indicates stronger connectivity for left seeds (i.e., left IFG, pSTG), while the blue color bar indicates stronger connectivity for right seeds (i.e., right IFG, pSTG). L, left hemisphere; R, right hemisphere.

Among the examined ROIs, functional connectivity of pSTG in both age groups and of IFG in 3-year-olds showed significant hemispheric asymmetry. Asymmetric connectivity with ipsilateral and contralateral hemispheres was obtainable from left and right hemispheres separately (Fig 4). In general, 3-year-olds demonstrated stronger contralateral connectivity than 5-year-olds. In 3-year-olds, only middle cingulate cortex (MCC) showed significant ipsilateral connectivity with pSTG, and significant asymmetric FC of pSTG with its contralateral hemisphere was found in the superior parietal lobe (SPL)/superior occipital gyrus and MCC (see Table 3). In 5-year-olds, no cortical regions showed stronger ipsilateral hemispheric asymmetry with pSTG, while significant contralateral hemispheric connectivity with pSTG was observed in middle/superior temporal gyrus (MTG/STG) extending to supramarginal gyrus. Moreover, for 3-year-olds, two regions showed stronger ipsilateral connectivity with IFG, including supplementary motor area (SMA) extending to MCC and angular gyrus; IFG showed significant asymmetric FC with its contralateral hemisphere in IFG, superior frontal gyrus (SFG) and MTG/STG (Table 3). No other significant hemispheric asymmetry was observed in either group.

**Table 3:** Brain regions showing significant hemispheric asymmetry of functional connectivity with pSTG and IFG.

No.	Regions	BA	MNI coordinates			Peak Z value	Cluster size (voxels)
			x	y	z		
***i pSTG in 3-year-olds***							
**1**	R. superior parietal lobe/superior occipital gyrus	7	24	-66	45	3.96	155
**2**	L/R. middle cingulate cortex	31	6	-33	45	-5.16	200
***ii pSTG in 5-year-olds***							
**1**	R. middle/superior temporal gyrus extending to supramarginal gyrus		45	-15	-12	-4.84	378
***iii IFG in 3-year-old***							
**1**	R. inferior frontal gyrus	44/45	57	21	27	4.55	662
**2**	R. superior frontal gyrus	6/8	21	18	66	4.25	288
**3**	L. supplementary motor area extending to middle cingulate cortex	6	-15	3	48	4.65	175
**4**	R. middle/superior temporal gyrus	22/21	68	-26	2	-4.59	247
**5**	L. angular gyrus	39	-42	-57	24	-3.99	230

Note: BA, Brodmann area; L., left hemisphere; R., right hemisphere.

**Fig 5:**
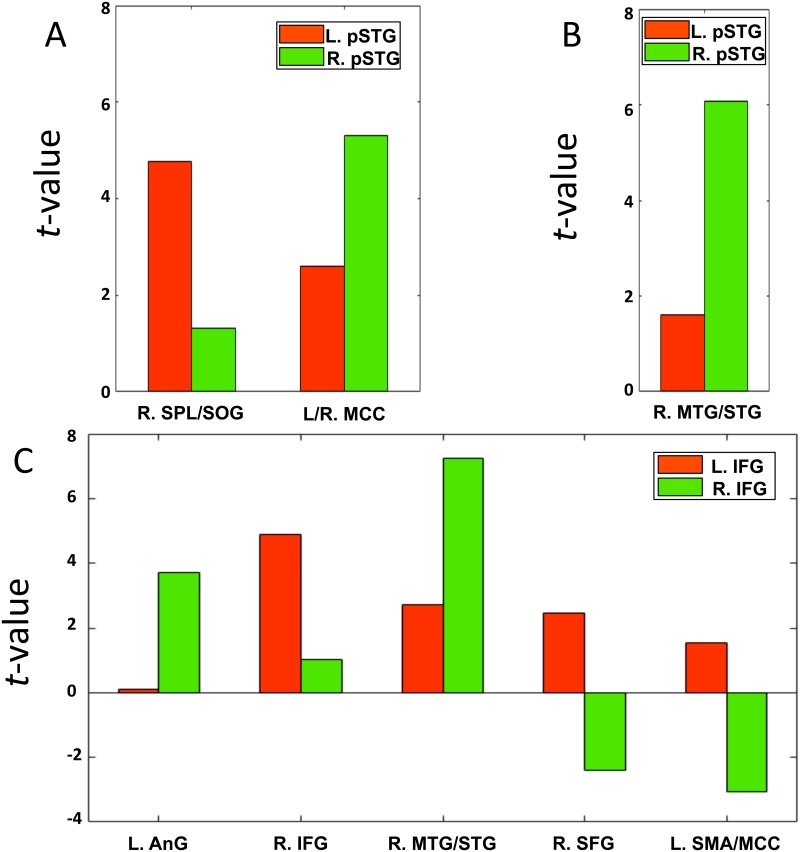
Average connectivity strength in the regions with significant functional hemispheric asymmetry of pSTG (3-year-olds, A; 5-year-olds, B) and of IFG (3-year-olds, C). The red color indicates connectivity strength calculated when seeded in the left pSTG/IFG; the blue color indicates connectivity strength calculated when seeded in the right pSTG/IFG. L., left hemisphere; R., right hemisphere; SPL, superior parietal lobe; SOG, superior occipital gyrus; MCC, middle cingulate cortex; MTG, middle temporal gyrus; STG, superior temporal gyrus; AnG, angular gyrus; IFG, inferior frontal gyrus; SFG, superior frontal gyrus; SMA, supplementary motor area. L, left hemisphere; R, right hemisphere.

In order to further evaluate the RSFC strength within each region that revealed significant hemispheric asymmetry, mean *t* values within each significant cluster (as shown in Table 3) were extracted from corresponding one-sample *t* maps of left ROIs (i.e., left IFG and left pSTG) and that of LR-flipped right ROIs (i.e., right IFG and right pSTG). The functional asymmetry patterns are shown in Fig 5. Specifically, connectivity strength with left pSTG and right pSTG in 3-year-olds was comparable albeit slightly stronger in left pSTG (a total average *t*-value of 7.36 and 6.62 for left and right IFG, respectively), while it showed greater RSFC strength with right pSTG than with left pSTG in 5-year-olds. In addition, three (ipsilateral SMA/MCC, contralateral IFG and SFG) out of five clusters showed greater connectivity strength with left IFG than right IFG in 3-year-olds.

**Fig 6:**
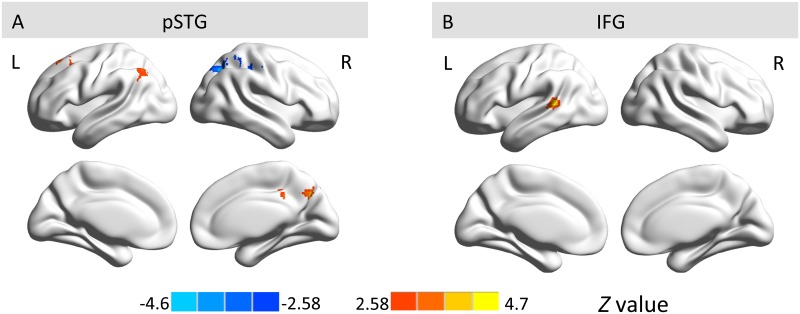
Group comparison of hemispheric asymmetry between 3- and 5-year-olds for pSTG (A) and IFG (B). The red-yellow color bar indicates regions with stronger functional asymmetry in 5-year-olds than 3-year-olds, while the blue color bar indicates regions with stronger functional asymmetry in 3-year-olds than 5-year-olds. L, left hemisphere; R, right hemisphere.

Furthermore, the group comparison for pSTG revealed increasing ipsilateral connectivity to SPL, angular gyrus, and MFG/SFG; it also demonstrated increasing contralateral connectivity to PCC and decreasing contralateral connectivity to SPL from ages 3 to 5 years (Fig 6 and Table 4). The IFG showed increasing ipsilateral connectivity to MTG/STG with age. No significant age difference was detected for aSTG.

**Table 4:** Group comparison of hemispheric asymmetry between 3- and 5-year-olds for pSTG and IFG.

No.	Regions	BA	MNI coordinates			Peak Z value	Cluster size (voxels)
			x	y	z		
***i pSTG***							
**1**	L. superior parietal lobe and angular gyrus	40	-54	-69	42	3.67	165
**2**	R. precuneus cortex	7	9	-69	36	3.99	152
**3**	L. middle/superior frontal gyrus	6	-3	45	54	3.59	149
**4**	R. superior parietal lobe		24	-66	45	-3.93	229
***ii IFG***							
**1**	L. middle/superior temporal gyrus	42/22	-54	-42	12	4.62	129

Note: The positive z value indicates greater hemispheric asymmetry in 5-year-olds compared to 3-year-olds. BA, Brodmann area; L., left hemisphere; R., right hemisphere.

## Discussion

In the present study, we employed a seed-based functional connectivity analysis to investigate the developmental changes of functional connectivity with brain regions belonging to the language network. Significant developmental changes were observed comparing preschool children at ages 3 and 5 years. We found stronger right aSTG RSFC in 3-year-olds than in 5-year-olds, and stronger connectivity between left IFG and left temporal cortex in 5-year-olds than in younger children when seeded in the left aSTG, pSTG and IFG. These results show that there is an increasing RSFC between left aSTG and left IFG, between left pSTG and left IFG, as well as between left IFG and left pSTS from ages 3 to 5 years. Moreover, functional hemispheric asymmetry analysis revealed that in 3-year-olds, RSFC of pSTG was almost bilateralized and RSFC of IFG showed leftward lateralization, while the RSFC of pSTG in 5-year-olds was mainly right lateralized. Both RSFC of IFG and of pSTG showed increasing left lateralization with age, but pSTG demonstrated a remaining dominance in rightward asymmetry at the age of 5 years. These findings suggest a clear developmental change of intrinsic brain connectivity in the language network in typically developing children towards a leftward lateralization.

### Lateralization of the language network and its development with age

The functional lateralization of the language network was examined in order to explore the process of the language lateralization with age. RSFC of pSTG was bilateral in 3-year-olds, but showed unilateral asymmetry in 5-year-olds. The right hemispheric asymmetry in 5-year-olds supports the notion of the engagement of the right hemisphere in language processing [14, 17, 49] and the finding of a temporal primacy in right hemispheric activation that can still be observed in 6-year-olds in contrast to adults [50]. Moreover, it is also consistent with previous studies showing right lateralization in Wernicke’s area in adults (i.e., pSTG/STS) [14, 21].

In contrast to the pSTG, RSFC of IFG demonstrated a trend of left lateralization in 3-year-olds and an increase of leftward asymmetry with age. This finding is not only in correspondence with previous rs-fMRI studies in adults that reported the left hemisphere dominance of the language network [14, 21], but also supported by language task fMRI studies in children [37, 38, 51, 52]. Notably, increasing connectivity between IFG and MTG/STG in the left hemisphere indicates an enhanced dorsal pathway in the brain of developing children [23, 25], which is crucial to language comprehension [16, 53]. This point is addressed in detail below.

However, neither significant hemispheric asymmetry in 3- and 5-year-olds, nor lateralization shift was observed in aSTG. Given the limited data in the present study, further research should shed light on this.

### Decreasing right aSTG connectivity from 3- to 5-year-olds

As discussed above, conclusion cannot be drawn from the present data that decreasing right aSTG connectivity is attributed to functional lateralization changes during this period, since no significant functional asymmetry in group comparison was observed. Therefore, we would provide an alternative interpretation for this finding.

Previous studies in adults have consistently reported activation of the right aSTG being involved in processing pitch information in tonal languages [54, 55], and this region was also activated in adult native Chinese speakers for pitch-related processing [39]. However, the study also unveiled greater activation in the right aSTG in English monolinguals due to their unfamiliarity with lexical tones rather than in native Chinese speakers [56]. It was further proposed that speech prosody perception is mediated primarily by the right-localized pitch processor and the left hemisphere comes into play when language processing goes beyond the auditory analysis, which is supported by other studies [57, 58]. These findings together suggest, concerning language processing, the function of right aSTG in relation to pitch perception and unfamiliar tone processing. Furthermore, behavioral studies in Chinese young children have shown evident improvement in tone perception from 2- to 5-year-olds [59] and significant developmental trend in speech recognition in children at ages of 3–6 years [60]. Therefore, we speculate that greater right aSTG connectivity in 3-year-olds is probably due to their unfamiliarity with lexical tones as marking lexical content and thereby requiring more resources for pitch processing. On the contrary, the decreasing right aSTG connectivity in 5-year-olds may benefit from an increase of familiarity with pitch as shown in previous behavioral studies [59, 60]. Right now this interpretation must remain and further empirical support is needed from both cross-culture studies and studies combined relevant behavioral tests in order to allow a solid conclusion.

### Increasing ventral connectivity between left aSTG and left IFG with age

The increasing RSFC between aSTG and IFG in the left hemisphere suggests an enhancement of the ventral processing stream from 3 to 5 years of age. The ventral pathway has been found to be related to sound-to-meaning integration [35], and its role in forming and accessing semantic representations makes it crucial to typical language development [61]. For instance, reduced activation in the ventral pathway predicts poor language ability in children with epilepsy [62]. A recent study, reviewing a number of language fMRI studies, reported increasing activations in the ventral pathway throughout childhood during semantic processing owing to increasing semantic completion within a growing lexicon and more semantic representations [61].

Therefore, in the present study, we infer that the increasing ventral pathway connectivity with age might be attributed to more demand of sound-to-meaning integration with increasing lexical and semantic input for children at the age of 5 years than children at the age of 3 years when less vocabulary is available. This is supported by a previous behavioral study which showed improvement in vocabulary and word recognition in Chinese preschool children from ages 3 to 6 years [63]. Increasing complex and expanding vocabulary probably demands more resources for semantic processing that is included in word identification through phonological information in Chinese. Thus, the findings here also support the notion that the stronger connection in the ventral pathway may be due to further semantic processing [39].

### Increasing dorsal connectivity between left pSTG/STS and left IFG with age

The current study also indicates age-related development of long-range functional connectivity between pSTG/STS and IFG in the left hemisphere, reflecting increasing connectivity in the dorsal pathway from ages 3 to 5 years. The finding of fronto-temporal functional connectivity can be related to the observation that the activation of left pSTG/STS has been consistently found to covary with IFG (i.e., BA 44) during processing syntactically complex sentences in language fMRI studies [22, 64–67]. It implies the role of the dorsal connection in the processing of sentential syntax with a non-canonical structure.

In Chinese, there is a non-canonical construction (i.e., the ‘bei’ construction), where the order of subject and object is reversed. The corresponding canonical construction is termed ‘ba’ construction with the normal order of subject and object. Apparently, the ‘bei’ construction should be more difficult to process than the ‘ba’ construction because it requires additional process for reversed word-order. Research has reported enhanced performance in response to the ‘bei’ construction during childhood [68, 69]. Chang [68] tested both ‘bei’ and ‘ba’ constructions in 3 to 5 year-old children, and observed a developmental trend with increasing performance in the ‘bei’ construction (44.4%, 56.9%, and 75% for 3-, 4- and 5-year-olds, respectively).

Therefore, we suppose that the increasing fronto-temporal connection in 5-year-old children might be related to the improvement in the processing of syntactic complexity.

### Limitations

A few limitations of the current study should be taken into account. Firstly, the interpretation of the results should be confined to -rs-fMRI context. The data shown here were from rs-fMRI rather than task-based fMRI experiment or LFFs analyses based on these. Secondly, a direct relation to language test was not available. Such data would have allowed for further conclusions about behavioral consequences of the observed functional network changes. Thirdly, rs-fMRI data were acquired when children were naturally sleeping. Natural sleep state might differ from regular resting state, but it has been widely used in previous pediatric studies (e.g., [9–11, 70]), and intrinsic functional connectivity appears to be stable in different consciousness states, even during sleep [71], or under anesthesia [72]. Lastly, the small sample size does not allow to test potential gender effects on the brain lateralization as shown in previous studies [73, 74].

Considering aforementioned limitations, future studies should include more children in each age group to enhance the robustness, and it is also suggested to investigate more age groups to sketch a complete picture of the developmental trajectory of the language network. Furthermore, it would be interesting to directly identify the relationship between changes in intrinsic brain connectivity and changes in language performance, or to obtain further evidence from task-related fMRI data. In addition, cross-language studies using the -rs-fMRI technique would be essential because it will open new horizons in revealing the similarities and dissimilarities of developmental trajectories of the language network when exposed to different language types (i.e., tonal and non-tonal languages).

## Conclusion

Our data here provide primary evidence for the development and lateralization of the language network in preschool children from task-free fMRI data. We found increasing left language lateralization in IFG from 3- to 5-year-olds and rightward asymmetry for pSTG in 5-year-olds. Moreover, we showed decreasing right aSTG RSFC, and observed greater connections in the ventral pathway between left aSTG and left IFG and increasing connectivity in the dorsal pathway between left IFG and left pSTG/STS in children from ages 3 to 5 years. These results suggest developmental trajectories of intrinsic brain connectivity in the language network. Notably, the present study must leave open whether the decreasing right aSTG RSFC and increasing fronto-temporal connection in the left hemisphere are attributed to the development of language abilities in general, or to specific abilities required by Chinese children and the language they learn. Nevertheless, these findings provide a first basis for cross-language comparisons with children from the different language (i.e., non-tonal language) environment in future research.
